# Supplementary material for: Nanoscopy reveals heparan sulfate clusters as docking sites for SARS-CoV-2 attachment and entry
Source: eLife. 2026 Aug 6;14:RP108925. doi: 10.7554/eLife.108925 (PMC13446900; doi:10.7554/eLife.108925)
Supplement: Peer review history

# Editor report: eLife Assessment

**DOI:** 10.7554/eLife.108925.3.sa0

**Authors:** John W Schoggins

**Affiliations:** The University of Texas Southwestern Medical Center United States

**Keywords:** Solid, Valuable

The authors use **solid** high resolution microscopy techniques to present a model in which SARS-CoV-2 attachment and endocytosis are mediated by heparan sulfate, whereas ACE2 only functions downstream of these early processes. These findings serve as a potentially **valuable** starting point to examine SARS-CoV-2 entry models that challenge current paradigms. However, examination of the model in a clean heparan sulfate deficient background and in the context of TMPRSS2-dependent plasma membrane fusion are lacking. Thus, the broader impact remains uncertain in the absence of additional controls and orthogonal approaches.

# Referee report: Reviewer #1 (Public review)

**DOI:** 10.7554/eLife.108925.3.sa1

This revised paper investigates how heparan sulfate (HS) engagement functions in the cellular entry of SARS-CoV-2. The authors used a series of microscopy techniques, labeled pseudoviruses and authentic SARS-CoV-2 strains, and cells lacking or expressing HS and/or hACE2 to re-examine the specific stage(s) HS and hACE2 function in the entry process. They suggest that HS mediates SARS-CoV-2 cell-surface attachment and endocytosis, and that hACE2 functions downstream of this to facilitate productive infection. Their results also suggest that SARS-CoV-2 binds clusters of HS molecules projecting 60-410 nm, which act as docking sites for viral attachment. The authors conclude their work establishes a revised entry paradigm in which HS clusters mediate SARS-CoV-2 attachment and endocytosis, with ACE2 acting at some stage downstream. They speculate this idea might apply broadly to other viruses known to engage HS and has translational implications for developing antiviral agents that target HS interactions.

The strengths of the study include the use of multiple high-resolution microscopy modalities, the tracking of labelled viruses, the use of both pseudoviruses and authentic SARS-CoV-2, and use of primary airway cells. While some studies were performed in the revision to address the Reviewer concerns, which improved the paper clarity, others were cursorily addressed, which limit the impact of the studies. Particularly. experiments were not performed to account for TMPRSS2 expression and plasma membrane fusion. Moreover, addition of studies in which hACE2 is expressed in cells genetically lacking HS were not designed. Thus, it the picture remains unclear picture exactly where downstream hACE2 functions and how this might differ given new structural models of TMPRSS2 activation (PMID: 42050172), which occur after ACE2 recognition of spike on the cell surface.

# Referee report: Reviewer #2 (Public review)

**DOI:** 10.7554/eLife.108925.3.sa2

In the manuscript by Han et al, the authors assess binding of SARS-CoV-2 to heparan sulfate clusters via advanced light microscopy of viral particles. The authors claim that SARS-CoV-2 spike (on the context of pseudovirus and in authentic virus) engages heparan sulfate clusters on the cell surface which then promotes endocytosis and subsequent infection. The finding that HSPGs are important for SARS-CoV-2 entry in some cell types is well described but the authors here attempt to make the claim that HS represents an alternative "receptor" and that HS engagement is far more important than the field appreciates. The data itself appears of appropriate quality and would be of interest to the field, but the overly generalized conclusions lack adequate experimental support. This significantly diminishes enthusiasm for this manuscript as written. Additional controls would be of great benefit.

Further, it is this reviewers opinion that the findings do not represent a novel paradigm as claimed. HS has been well described for SARS-CoV-2 and other viruses to serve as attachment factors to promote initial virus attachment. A more balanced and nuanced view of their interesting data would be of value.

Major:

The authors need to rigorously define a "receptor." This reviewer would argue that a receptor is a host factor that is necessary and sufficient for active promotion of viral entry (genome release into the cytoplasm) while an attachment factor is a host factor that enhances initial viral attachment/endocytosis but is not necessary nor sufficient. The evidence does NOT implicate HS as a receptor under this definition. This is proven in Fig 1 (and elsewhere) in which ACE2 is absolutely required for viral entry.

The authors should genetically perturb HS biosynthesis in their key assays to demonstrate necessity. HS biosynthesis genes have been shown to be important for SARS-CoV-2 entry into some cells but not others Huh7.5 cells PMID 33306959 but not in Vero cells PMID 33147444, Calu3 cells 35879413, A549 cells 33574281, and others 36597481. This is inconsistent with the claim that HS is broadly important (beyond the BHK cells overexpressing ACE2 that are used here).

Is targeting HS really a compelling anti-viral strategy? The data show a ~5-fold reduction. The strengths and limitations of HS targeting should be presented in a more balanced discussion. Animal data showing anti-viral activity of PIX is warranted. This would enhance this claim and also provide key evidence of a relevant role for HS in a more physiologic model.

The authors provide inadequate discussion into the fact that these studies rely exclusively on cell lines (which also happen to be TMPRSS2 deficient). The role of proteases in the role of HS should be tested in the cell lines and primary cells used as protease expression is a key determinant of the site of fusion.

An alternative method to disrupt HS (other than PIX) is needed in primary airway cells. A genetic approach would be much more convincing. The authors should also demonstrate whether entry in their primary cell assays are TMPRSS2 vs Cathepsin L dependent (using E64d and camostat for instance) as mentioned above.

Each figure legend should clearly state how many independent experiments and replicates per experiment were performed.

All bar plots should show individual dots (i.e. Fig 1G) to better reveal the variance of each dataset.

# Author comment: Author response

**DOI:** 10.7554/eLife.108925.3.sa3

**Authors:** Sue Han, Xin Wang, Tiansheng Li, Ammar Mohseni, Ivan Kosik, Chuang Yu Chan, Alberto Domingo López-Muñoz, Jessica Matthias, Reid Suddaby, Zhixiong Wang, Albert J Jin, Christian A Wurm, Jonathan W Yewdell, Ling-Gang Wu

**Affiliations:** National Institute of Neurological Disorders and Stroke Bethesda United States; National Institute of Neurological Disorders and Stroke Bethesda United States; National Institute of Allergy and Infectious Diseases Bethesda United States; National Institute of Neurological Disorders and Stroke Bethesda United States; National Institute of Allergy and Infectious Diseases Bethesda United States; National Institute of Neurological Disorders and Stroke Bethesda United States; National Institute of Allergy and Infectious Diseases Bethesda United States; Abberior instruments America LLC Bethesda United States; National Institute of Neurological Disorders and Stroke Bethesda United States; National Institute of Biomedical Imaging and Bioengineering Bethesda United States; National Institute of Biomedical Imaging and Bioengineering Bethesda United States; Abbeiror Instruments America, LLC Bethesda United States; National Institute of Allergy and Infectious Diseases Bethesda United States; National Institute of Health Bethesda United States

The following is the authors’ response to the original reviews.

**Public Reviews:**

**Reviewer #1 (Public review):**

This paper investigates how heparan sulfate (HS) engagement functions in the cellular entry of SARS-CoV-2. A prevailing model that has been developed over the last five years by work from many laboratories using a variety of biochemical, structural, and microscopic approaches is that HS acts a co-receptor for SARS-CoV-2; its binding to SARS-CoV-2 both concentrates virus on the surface of target cells and allosterically alters the spike protein to promote an "up/open" RBD conformation that enables engagement of the proteinaceous receptor human ACE2 on the cell surface (PMID: 32970989, 35926454, 38055954, 39401361, 40548749). These two events enable plasma membrane fusion (after a cleavage event promoted by plasma membrane TMPSS2) or endocytosis and subsequent pH-dependent fusion (which requires a cathepsin L-mediated cleavage of the spike).

The authors in this study used a series of microscopy techniques, labeled pseudoviruses and authentic SARS-CoV-2 strains, and cells lacking or expressing HS and/or hACE2 to re-examine the specific stage(s) HS and hACE2 function in the entry process. They suggest that HS mediates SARS-CoV-2 cell-surface attachment and endocytosis, and that hACE2 functions "downstream" of this to facilitate productive infection. Their results also suggest that SARS-CoV-2 binds clusters of HS molecules projecting 60-410 nm, which act as docking sites for viral attachment. Blocking HS binding with pixantrone, a drug under clinical evaluation for cancer (due to its anti-topoisomerase II activity), inhibited SARS-CoV-2 Omicron JN.1 variant from attaching to and infecting human airway cells. The authors conclude that their work establishes a revised entry paradigm in which HS clusters mediate SARS-CoV-2 attachment and endocytosis, with ACE2 acting at some stage downstream. They speculate this idea might apply broadly to other viruses known to engage HS and has translational implications for developing antiviral agents that target HS interactions.

The strengths of the interesting and technically well-executed study include the use of multiple high-resolution microscopy modalities, the tracking of labelled viruses, the use of both pseudoviruses and authentic SARS-CoV-2, and the use of primary airway cells. Nonetheless, there are issues that need to be addressed to buttress the proposed model compared to earlier ones. These include: (a) the distinction between macropinocytosis and receptor-mediated endocytosis and what this might mean for productive SARS-CoV-2 infection; (b) the need to account for TMPRSS2 expression and plasma membrane fusion; (c) addition of genetic studies in which hACE2 is expressed in cells lacking HS; (d) an unclear picture of exactly where downstream hACE2 functions; and (e) and a need for comparative/additional study of earlier SARS-CoV-2 variants, which preferentially fuse at the plasma membrane.

We thank the reviewer for the strong support of this manuscript. We addressed the reviewer’s concerns in the Recommendations to the authors. We did not distinguish whether the endocytic route is macropinocytosis or receptor-mediated endocytosis, because it is a separate study beyond the scope of the present work. We did not examine earlier SARS-CoV-2 variants because we considered it a study beyond the scope of the present work, but a good idea that we may work on in the future. For detail on how we address the remaining concerns, please see our response to the reviewer’s Recommendations for the authors.

**Reviewer #2 (Public review):**

In this manuscript by Han et al, the authors assess the binding of SARS-CoV-2 to heparan sulfate clusters via advanced light microscopy of viral particles. The authors claim that the SARS-CoV-2 spike (in the context of pseudovirus and in authentic virus) engages heparan sulfate clusters on the cell surface, which then promotes endocytosis and subsequent infection. The finding that HSPGs are important for SARS-CoV-2 entry in some cell types is well-described, but the authors attempt to make the claim here that HS represents an alternative "receptor" and that HS engagement is far more important than the field appreciates. The data itself appears to be of appropriate quality and would be of interest to the field, but the overly generalized conclusions lack adequate experimental support. This significantly diminishes enthusiasm for this manuscript as written. The manuscript is imprecise and far overstates the actual findings shown by the data. Additional controls would be of great benefit.

Further, it is this reviewer's opinion that the findings do not represent a novel paradigm as claimed. HS has been well described for SARS-CoV-2 and other viruses to serve as attachment factors to promote initial virus attachment. While the manuscript provides new insight into the details of this process, the manuscript attempts to oversell this finding by applying new words rather than new molecular details. The authors would be better served by presenting a more balanced and nuanced view of their interesting data. In this reviewer's opinion, the salesmanship significantly detracts from the data and manuscript.

We thank the reviewer for pointing out that our manuscript is of interest to the field. However, we do not think that we oversell our data. hACE2 has been widely considered the receptor (or the binding partner) that mediates SARS-CoV-2 cell-surface attachment, whereas HS is considered only an attachment factor that facilitates SARS-CoV-2 binding with hACE2 at the cell surface. In the present work, we found that HS, but not hACE2, is the cell-surface attachment receptor (or binding partner), whereas hACE2 is not essential for attachment, but acts downstream of virus endocytosis to facilitate viral genome expression. This finding suggests significant modification of the current model by replacing the attachment receptor (or binding partner) from hACE2 to HS, treating HS as a primary receptor rather than an attachment factor, and relocating the hACE2 action site from the cell surface to the endosome. For these reasons, we do not consider these statements overselling our data. However, as the reviewer suggested in his/her specific comments, we revised the manuscript to ensure that we did not overgeneralize our findings (see our responses to the reviewer’s Recommendations to the authors).

Major Comments:

The authors need to rigorously define a "receptor" vs an "attachment factor." They also should avoid ambiguous terms such as "receptor underlying ...attachment" and "attachment receptor" (or at least clearly define them). Much of their argument hinges on the specific definition of these terms. This reviewer would argue that a receptor is a host factor that is necessary and sufficient for active promotion of viral entry (genome release into the cytoplasm), while an attachment factor is a host factor that enhances initial viral attachment/endocytosis but is neither necessary nor sufficient. The evidence does NOT implicate HS as a receptor under this fairly textbook definition. This is proven in Figure 1 (and elsewhere) in which ACE2 is absolutely required for viral entry.

The authors should genetically perturb HS biosynthesis in their key assays to demonstrate necessity. HS biosynthesis genes have been shown to be important for SARS-CoV-2 entry into some cells but not others Huh7.5 cells PMID 33306959, but not in Vero cells PMID 33147444, Calu3 cells 35879413, A549 cells 33574281, and others 36597481. The authors need to discuss this important information and reconcile it with their data and model if they want to claim that HS is broadly important.

Is targeting HS really a compelling anti-viral strategy? The data show a ~5-fold reduction, which likely won't excite a drug company. The strengths and limitations of HS targeting should be presented in a more balanced discussion. Animal data showing anti-viral activity of PIX is warranted. This would enhance this claim and also provide key evidence of a relevant role for HS in a more physiologic model.

The authors provide little discussion of the fact that these studies rely exclusively on cell lines (which also happen to be TMPRSS2-deficient). The role of proteases in the role of HS should be tested in the cell lines and primary cells used, as protease expression is a key determinant of the site of fusion.

The claim that "SARS-CoV2 JN.1 variant binds to heparan sulfate, not hACE2, in primary human airway cells" is extraordinary and thus requires extraordinary evidence.

First, PIX reduces attachment by 5-fold, which is not the same as "nearly abolished." Also, anti-ACE2 "nearly abolished" entry in 7D, while PIX did not. If the authors want to make these claims, an alternative method to disrupt HS (other than PIX) is needed in primary airway cells. A genetic approach would be much more convincing. The authors should also demonstrate whether entry in their primary cell assays is TMPRSS2 vs Cathepsin L dependent (using E64d and camostat, for instance) as mentioned above.

Each figure should clearly state how many independent experiments and replicates per experiment were performed. What does "3 experiments" mean? Are these three independent experiments or three wells on one day?

In the well-accepted current model, hACE2 is considered the receptor mediating SARS-CoV-2 cell-surface attachment, entry into cells, and infection, whereas HS is an attachment factor that facilitates SARS-CoV-2 binding to hACE2 at the cell surface. The present work revises this view: HS is the SARS-CoV-2 attachment receptor mediating virus docking at the cell surface, with ACE2 acting downstream of virus endocytosis to enable SARS-CoV-2 infection in the cell types examined.

We made this point clearer throughout the newly revised manuscript. We define the attachment receptor as the docking site where the virus binds to the cell surface. We directly showed with several super-resolution imaging techniques that the virus docks at HS clusters.

The cited CRISPR-screen literature supports context-dependent host-factor usage. However, the absence of HS biosynthesis genes from a given screen does not prove that HS is irrelevant in that cell type; it only indicates that HS biosynthesis was not detected as a genetic dependency under that assay’s conditions. Such negative results can reflect screen sensitivity, incomplete knockout, pathway redundancy, or viral dose/stringency. In the revised manuscript, we included the following in the Discussion:

“While some studies using genome-wide CRISPR screening to identify genes involved in SARS-CoV-2 reveal genes for HS biosynthesis, others do not (45-50). The negative result, which might reflect screen sensitivity, incomplete knockout, pathway redundancy, or viral dose/stringency, needs to be verified with specific gene knockout.”

The ~5-fold reduction is likely due to the inhibitor not completely abolishing HS-virus binding. We revised the Discussion to strengthen the suggestion that targeting the virus cell-surface attachment by interfering HS binding is a therapeutic strategy to prevent and treat COVID-19, as in the following:

“Interfering with HS binding has been suggested as a therapeutic strategy to prevent and treat many viral infections that depend on HS for entry, including COVID-19 [1, 2, 9, 12]. Supporting this strategy, disrupting Spike–HS interactions, including inhibition by heparin and related glycans, reduces SARS-CoV-2 attachment/entry [51]. Clinical evaluation of inhaled/nebulized unfractionated heparin has reported improved clinical outcomes without major bleeding signals, supporting the feasibility of targeting airway-surface HS interactions [52]. HS mimetics, such as pixatimod (PG545), inhibit SARS-CoV-2 infection and exhibit greater potency than heparin in assays measuring inhibition of Spike/ACE2 engagement and viral infectivity [53]. These reports support the translational potential of therapeutically interfering with virion–HS binding. However, this strategy has not been the focus for developing methods to prevent and treat COVID-19, likely because HS is considered only a regulator that is not essential for SARS-COV-2 entry. Our finding that HS is the attachment receptor re-emphasizes the importance of perturbing virus-HS binding, the first step of the viral entry, to efficiently block SARS-CoV-2 infection. Further supporting this view, inhibition of HS binding with a clinically used HS-binding agent, pixantrone, inhibits authentic SARS-CoV-2 JN.1 subvariant binding with HS on the cell surface and infection in primary human airway cells (Figs. 6, 7). These results suggest a combinatorial anti-SARS-CoV-2 strategy: early HS blockade to prevent attachment combined with ACE2 targeting to inhibit post-attachment steps”

We include a sentence in the Discussion that our suggestions are limited to the cells we examined as below.

“For other cells not examined in the present work, if TMPRSS2 is highly expressed, we could not rule out the possibility that the fusion pathway could also be dominant.”

Three experiments refer to three independent experiments. We added “independent” accordingly throughout the manuscript.

**Reviewer #3 (Public review):**

Summary:

In this manuscript, the authors define a new paradigm for the attachment and endocytosis of SARS-CoV-2 in which cell surface heparan sulfate (HS) is the primary receptor, with ACE2 having a downstream role within endocytic vesicles. This has implications for the importance of targeting virion-HS interactions as a therapeutic strategy.

Strengths:

The authors show that viruses are internalized via dynamin-dependent endocytosis and that endocytic internalization is the major pathway for pseudotyped SARS-CoV-2 genome expression. They show that HS-mediated viral attachment is a critical step preceding viral endocytosis and also subsequent genome expression. Further, they show that hACE2 acts downstream of endocytosis to promote viral infection, and may be co-internalised with virions after HS attachment. Pseudotyped virus and authentic SARS-CoV-2 provide similar results. In addition, the authors demonstrate that remarkable clusters of multiple HS chains exist on the cell surface, visualised by a number of elegant microscopy methods, and that these represent the docking sites for virions. These visualisations are an important general contribution in themselves to understanding the nanoscale interactions of HS at the cell surface.

The use of a complementary range of methods, virus constructs, and cell models is a strength, and the results clearly support the conclusions.

Overall, the results convincingly demonstrate a different model to the currently accepted mechanism in which the ACE2 protein is regarded as the cell surface receptor for SARS-CoV-2. Here, the authors provide compelling evidence that cell surface clusters of HS are the primary docking site, with ACE2 interactions occurring later, after endocytosis (whilst still being essential for viral genome expression). This is an exciting and important landmark evidence which supports the view that HS-virion interactions should be viewed as a key site for anti-viral drug targeting, likely in strategies that also target the downstream ACE2-based mechanism of viral entry within endosomes.

We thank the reviewer for the strong support of the present work.

Weaknesses:

This reviewer identified only minor points regarding citing and discussing other studies and typos, which can be corrected.

We have addressed these points in the revised manuscript. For detail, please see our response to the reviewer’s Recommendations to the authors.

**Recommendations for the authors:**

**Reviewer #1 (Recommendations for the authors):**

(1) Pathway of internalization.

The authors show clearly that labeled SARS-CoV-2 (pseudovirus or authentic virus) can become internalized in cells lacking hACE2, and this process depends on HS. However, they also show that this pathway is non-productive with regard to infection. Are the entry vesicles mediated by HS alone, HS + hACE2, and hACE2 alone the same? Or does the combination of co-receptor (HS + hACE2) drive SARS-CoV-2 into endocytic vesicles, whereas HS alone promotes macro- or micropinocytosis (lines 361-362). If HS alone directed SARS-CoV-2 into a non-productive entry vesicle, then hACE2 likely would be acting concurrently with HS and not downstream. A more detailed analysis of the different entry vesicles/pathways that occur with HS alone, HS + hACE2, and hACE2 alone is needed.

During endocytosis, we did not detect a difference in the size distribution of virus-containing vesicles between BHK (HS alone) and BHK_hACE2_ cells (HS+hACE2) (Fig. 2D). The similarity in the vesicle size suggests a similar endocytic path with HS alone or with HS + hACE2. In the revised manuscript, we added the following sentence.

“Third, 3D-STED imaging showed that A490-labeled vesicle’s full-width-at-half-maximum (W_H_) was 363 ± 17 nm (n = 55) in BHK cells, similar to that (333 ± 13 nm, n = 70) in BHK_hACE2_ cells (Fig. 2B-D), supporting a similar endocytic path regardless of hACE2 presence or not.”

(2) TMPRSS2 and plasma membrane fusion.

Although the authors allude to membrane fusion as an alternate mechanism of entry, their mechanistic experiments do not address the roles of HS and hACE2 in this process, possibly because their BHK and other cells do not co-express significant levels of TMPRSS2. While many Omicron variants preferentially enter cells via endocytosis (relative to antecedent strains in the pandemic) because of spike mutations that reduce cleavage by TMPRSS2 (PMID: 35104837, 36625591, 35145066), plasma membrane fusion can still occur. The authors should add experiments with co-expression of TMPRSS2/hACE2 [with or without HS] and earlier SARS-CoV-2 variants to establish the role of HS in plasma membrane fusion. Also, are there differences in entry pathways if viruses are prepared in cells expressing TMPRSS2?

We thank the reviewer for this important comment and agree that our mechanistic experiments were not designed to address TMPRSS2-supported plasma membrane fusion. The reviewer’s suggestion for direct testing of HS function in TMPRSS2-supported plasma membrane fusion, including hACE2/TMPRSS2 co-expression and comparison with earlier SARS-CoV-2 variants, will require dedicated experiments and detection of the fusion pathway that we have not yet designed. It is beyond the scope of the present work. In the revised manuscript, we clarify that the observed ACE2-independent uptake and the predominance of endocytic entry refer to the tested cell systems and do not exclude TMPRSS2-dependent plasma membrane fusion in other cell types, as in the following.

“For other cells not examined in the present work, if TMPRSS2 is highly expressed, we could not rule out the possibility that the fusion pathway could also be dominant.”

(3) Experiments with hACE2 in cells lacking HS.

Apart from drug treatment (heparinases or pixantrone) studies shown, the current studies do not directly address whether expression of hACE2 on human cells can allow for endocytosis and productive infection in the complete and genetic absence of HS. The only experiments that use genetically deficient cells are the CHO [hamster] cell studies, and these cells lack hACE2 expression. The authors should knock out a key HS biosynthesis gene (e.g., B4GALT7) in more relevant human cells (e.g., A549-hACE2; ideally with sorted subpopulations having different levels of surface hACE2 expression) and assess endocytosis and infection. This is important given studies in the literature by others suggesting that KO of HS expression reduces but does not abrogate SARS-CoV-2 infection.

We thank the reviewer for these comments. We showed that virus endocytosis is independent of hACE2 (Fig. 1). The reviewer’s question is whether hACE2 alone can allow for endocytosis of viruses. We have shown that in either BHK (without hACE2) or BHK_hACE2_ cells (BHK cells expressed with hACE2), heparinase I/II/III mixture (HPRase) nearly abolished cell-surface immunolabelled HS (Fig. 3D), reduced cell-surface virus attachment by ~83-85% (Fig. 3E), reduced viral uptake by ~80% (Fig. 3F, 3G). These results suggest that hACE2 is not essential for viral attachment and endocytosis. We did not test whether hACE2 alone (without HS) plays a minor role for viral attachment and endocytosis, because to our knowledge, HS is present in nearly every cell. Under this physiological condition, it is HS, not hACE2, that plays an essential role in viral cell-surface attachment and endocytosis. In the revised manuscript, we added a sentence admitting that we did not test whether hACE2 alone is sufficient to support viral uptake and productive infection, as in the following.

“Our data suggest that, under physiological conditions in which HS is present at the cell surface, hACE2 is not essential for viral cell-surface attachment or endocytosis. We do not know whether hACE2 expression alone, in the absence of HS, can support viral cell-surface attachment and endocytosis.

(4) hACE2 function in entry.

In many places, the authors suggest that hACE2-spike functional interaction occurs "downstream" of HS-dependent binding and endocytosis (e.g., lines 25, 33, 48, 210, 309, 312, 318, 333, 346). However, in their model, it is not clear where exactly this interaction occurs. Are the authors suggesting that this spike binds hACE2 on the cell surface, but this has nothing to do with endocytosis, or that the interaction with hACE2 is occurring at a post-entry step? Can they experimentally demonstrate the stage at which hACE2 is functioning? Is it the same or different in cells lacking HS? What about when TMPRSS2 is present?

We showed that viral attachment and endocytosis are independent of hACE2, whereas entry as determined by viral gene expression, depends on hACE2. We also showed that most virions bind to HS, not hACE on the cell surface. Based on these results, we propose a model that hACE2 functions downstream of virion endocytosis. We cannot rule out the possibility that a small subset of viruses can also bind to hACE2 after their binding with HS at the cell surface.

We have not been able to design an experiment to visualize hACE2 mediated virion fusion in endosomes, where hACE2 may facilitate virus fusion and delivery of viral genomes to the cytosol. Productive infection requires only a limited number of successful virion–hACE2 engagement events. While many internalized virions can be visualized, the specific virion or vesicle that ultimately gives rise to productive infection cannot be identified from the present imaging data. This makes it difficult to trace the precise stage or compartment in which the functionally relevant spike–hACE2 interaction occurs. In the revised manuscript, we added a paragraph discussing this limitation as below.

“Our data suggest that, under physiological conditions in which HS is present at the cell surface, hACE2 is not essential for viral cell-surface attachment or endocytosis. We do not know whether hACE2 expression alone, in the absence of HS, can support viral cell-surface attachment and endocytosis. Although our data suggest that hACE2 functions downstream of endocytosis to facilitate viral fusion at the endosome for genome delivery to the cytosol, we do not know whether hACE2 binding with the virus occurs at the cell surface or endosomes. The binding may occur in both places, but not essential for virus attachment and endocytosis.”

(5) Other comments.

(a) Figure 1A. "Antibody" is misspelled.

Corrected. Thank you.

(b) The imaging experiments with pseudoviruses and authentic viruses lack any information on the multiplicity of infection or the number of virions added per cell. If this is particularly high and non-physiological (e.g., >100), is it possible that such conditions might enable viruses to enter [dominantly] through secondary [non-infectious] pathways?

To address the reviewer’s concern, we used flow cytometry to measure cell-associated VSV-S signal as we diluted the virus by ~600-fold. We found that the V-A647 attachment at the cell surface of BHK cells was similar to that in BHK_hACE2_ cells over a ~600-fold dilution of the virus (Fig. S7), indicating that the virus cell-surface attachment is independent of hACE2 across a wide range of virus concentrations. In the revised manuscript, we included the following sentence and Fig. S7 (Supplementary Information).

“Flow cytometry also showed that the V-A647 attachment at the cell surface of BHK cells was similar to that in BHKhACE2 cells over a ~600-fold dilution of the virus concentration (Fig. S7), indicating that the virus cell-surface attachment is independent of hACE2 across a wide range of virus concentrations.”

(c) Figure 1C and elsewhere. Most of the internalization studies rely on various imaging modalities to demonstrate the pseudovirus or virus on or in the cell. The experiments would be strengthened by inclusion of data from orthogonal binding/internalization assays that measuring virion-associated viral RNA on the surface [4oC binding assay] or inside the cell [after a 37oC temperature shift and exogenous proteinase K and RNAse A treatment] - such assays can be performed at much lower MOI (e.g., <1, addressed comment #2 above) an also allow more objective quantitation and kinetic analyses of virus internalization (e.g., 0, 5, 15, 30 min at 37oC).

We demonstrate virion attachment and uptake using multiple approaches, including confocal, STED, and EM analysis, showing virions with the expected morphology at the cell surface and in the cytosol. Furthermore, flow cytometric analysis provides population-level quantitation supporting the same overall conclusion. Thus, while we appreciate and agree that an RNA-based binding/internalization assay would provide additional information, we do not consider it essential to the main conclusion of this work.

(d) Figure 2. (i) Is there any indication of which vesicles the bath dye is in? Is most of this fluid taken up by micropinocytosis? Are these the same vesicles where the virus that is destined for productive infection (HS/hACE2 engaging) transits? (ii) In all panels, can the authors clearly indicate/label which cells are being used (BHK or BHK-hACE2)? (iii) For the studies with dynasore or dominant-negative dynamin-2-K44A, the readout is at 24 h, a late timepoint, which also could affect virus egress and spread. Can the studies be repeated at much earlier time points (e.g., 15 min to 2 h) to demonstrate that viruses are internalized via dynamin-dependent endocytosis in these cells?

(i) The bath dye A490 was used as a fluid-phase marker for endocytic uptake, rather than as a marker for a specific vesicle class or intracellular compartment. In principle, any vesicle that takes up extracellular fluid could become labelled by this approach. Since nearly all viruses are in the A490-containing vesicles, productive virus infection must come from some of these vesicles.

(ii) In the revised Fig. 2 legends, we explicitly indicate which cells are used for each panel.

(iii) To address the reviewer’s concern, we examined earlier time points for dynasore treatment and found that the virus uptake and genome expression were already markedly reduced at 1 h and 8 h after virus incubation. In the revised manuscript, we described these results as below and in Fig. S5.

“Fourth, dynasore or dominant-negative dynamin 2-K44A overexpression, which inhibits fission of dynamin-dependent endocytosis [28-30], substantially reduced V-A647 internalized 1-24 h after viral incubation (Figs. 2F-G, S5).

In addition to inhibiting V-A647 endocytosis, dynasore or dynamin 2-K44A inhibited *V-EGFP* expression 8-24 h after virus incubation by ~66-77% (Figs. 2F-G, S5), suggesting that endocytosis is the main route for viral genome expression.”

(e) Line 225. "Envelop" should be "envelope".

Corrected, thank you.

(f) Line 235. The authors should clarify that they conclude that the "Omicron variant" of SARS-CoV-2 enters "BHK" cells indistinguishably from VSV-S.

Thank you for pointing this out. We have rephrased the conclusion as “…omicron variant of SARS-CoV-2 enters BHK cells indistinguishably to VSV-S.”

(g) Line 278. What happens to virus binding if the authors ectopically express hACE2 in CHO-K1 WT and CHO-pgsA-745 cells?

We did not perform this experiment (see also our response to major comment 3 above).

(h) Lines 280-281 and elsewhere (line 635). The authors state "pixantrone (PIX), a drug under clinical trial that binds HS to inhibit HS binding with proteins...." The authors should clarify that the drug is under clinical evaluation for cancer treatment because of its DNA intercalating activity (and not its HS binding activity) and cite any relevant ongoing trials. Also, in line 635, is reference #46 correct?

As suggested, we modified this sentence as “pixantrone (PIX), a drug under clinical trial for cancer treatment due to its DNA intercalating activity, which can bind HS to inhibit HS binding with proteins”

(i) Line 281-282. The authors should confirm in a Supplementary Figure that the anti-hACE2 antibody used blocks SARS-CoV-2-JN.1 binding to ACE2.

In Figure 7D, we showed that PIX and anti-hACE2 antibody block SARS-CoV-2-JN.1 infection, suggesting that anti-hACE2 blocks SARS-CoV-2-JN.1 binding with hACE2.

(j) Figure 7B. Can hACE2 co-localization be added to this panel?

We did not perform this experiment. We addressed the role of ACE2 in these airway cells in subsequent panels of Fig. 7.

(k) Figure 7C. The quantitative data show a 50% reduction in binding signal with pixantrone, whereas the microscopy images appear to show a much greater effect. Can more representative images be shown so that the data better corresponds?

A ~50% effect is not as visually obvious as the current Fig. 7C. Therefore, we chose not to change the images. However, the statistics in Fig. 7C (right) clearly indicate an average effect of about 50%, as the reviewer pointed out.

(l) In the Discussion, it is not necessary to use Figure callouts (as done in the Results). Please remove, with the exception of reference to the model.

We prefer to call out Figures in the Discussion so that we can remind the readers where to find the data. The readers may choose to neglect these callouts. But some readers may read most the discussion part without going through the results carefully. In this case, the figure callouts may help these readers.

(m) Please delete all references to "new" or "novel" models. It is unnecessary.

As the reviewer suggested, we deleted “new” and “novel” throughout the revised manuscript.

(n) Figure legends. Please make sure each panel indicates the # of independent experiments performed. This is included for some but not all panels. Also, a few panels use an unpaired t-test where an ANOVA with multiple comparisons is required (e.g., Figure 1G and S1).

We agree that, for the three-group sub-comparisons shown within Fig. 1G and Fig. S1, the relevant analyses should account for multiple comparisons. In the revised manuscript, we therefore analyzed these predefined three-group subsets using ordinary one-way ANOVA followed by Dunnett’s multiple-comparisons test, with BHK or Vero used as the reference group as appropriate. The two-group comparisons were analyzed using unpaired two-tailed t-tests.

**Reviewer #2 (Recommendations for the authors):**

(1) It is well established that ACE2 is the receptor for SARS-CoV-2. The authors should not downplay this by saying it is "widely assumed", "typically thought", etc. The specific molecular details at various stages of entry (i.e, the role of HS) remain a bit unclear, but it is disingenuous to imply ACE2 is not the bona fide receptor by any conventional definition.

The present work does not challenge the well-established view that ACE2 is the receptor for SARS-CoV-2 entry/infection, but suggests that HS is the SARS-CoV-2 attachment receptor mediating virus docking at the cell surface, whereas ACE2 acts downstream of virus endocytosis to enable SARS-CoV-2 infection in the cell types examined. We made this point clearer throughout the revised manuscript. We define the attachment receptor as the docking site where the virus binds to the cell surface. We directly showed with several super-resolution imaging techniques that the virus docked at the HS clusters.

As the reviewer suggested, we removed “assumed” and “typical” and clarify that our findings do not challenge this concept. For example, we modified the abstract

“Virus entry is thought to involve binding a unique receptor for cell attachment and cytosolic entry. For SARS-CoV-2 underlying the COVID-19 pandemic, angiotensin-converting enzyme 2 (ACE2) is widely assumed as the receptor.”

as

“Virus entry is thought to involve binding a unique receptor for cell attachment and cytosolic entry. For SARS-CoV-2 underlying the COVID-19 pandemic, angiotensin-converting enzyme 2 (ACE2) is widely considered the receptor for cell-surface attachment and subsequent cell entry.”

(2) When the authors state pseudovirus internalization is independent of ACE2, they should clarify that this is the case in cells not expressing TMPRSS2. Most physiologically relevant cell types express TMPRSS2, which will facilitate entry at the plasma membrane.

As the reviewer suggested, we included the following sentence in the Discussion section: “For other cells not examined in the present work, if TMPRSS2 is highly expressed, we could not rule out the possibility that the fusion pathway could also be dominant.”

(3) Line 130: "Endocytic internalization is the main viral infection pathway" and Line 180-181 is not precise and should be rephrased to include the cell types described in the figure. This may be true in BHK-ACE2 cells, but the evidence in this section does not show that this is universally or broadly true.

We agree and have revised these sentences to limit the conclusions to the experimental context directly supported by our data. Specifically, our results support endocytic uptake as the major route leading to pseudovirus genome expression in the pseudovirus assays and cell types examined here, rather than as a universal entry mechanism for SARS-CoV-2 across cell types. We have therefore modified the subsection title and the relevant sentence in the Results to explicitly refer to the tested cells/assays.

Across the revised manuscript, we have accordingly revised the text to distinguish initial virion docking/attachment from productive entry, to acknowledge ACE2 as the established receptor for productive infection, and to limit our mechanistic conclusions to the cellular systems directly tested here.

(4) All bar plots should show individual dots (i.e., Figure 1G) to better reveal the variance of each dataset.

While we respect the reviewer’s suggestion, this is not required in the journal style. We prefer plotting bar graphs without individual data points, which often makes it difficult to see the mean values.

(5) Line 57: This is not accurate. HIV uses a receptor and a co-receptor, for instance.

We thank the reviewer for noting this inaccuracy. We agree that viral entry frequently involves coordinated engagement of multiple host factors rather than a single receptor, for example, HIV requires both a primary receptor and a co-receptor. We have revised the statement in the Introduction (Line 57–58) to reflect that entry can involve receptors together with co-receptors and/or attachment factors, which collectively facilitate membrane fusion or endocytic uptake.

In the Introduction (Line 57), we replaced the sentence with “Viral entry is often initiated by engagement of host receptors and associated co-factors that together facilitate subsequent viral membrane penetration.”

(6) Line 60: "most" --> "many"

As suggested, we have changed “most” to “many”.

(7) Remove "clinically relevant" in reference JN.1, as JN.1 is not circulating currently. A more appropriate term could be "full-length" or "authentic", or "wild-type".

As suggested, we changed it to “authentic”.

**Reviewer #3 (Recommendations for the authors):**

(1) The authors omit to mention the work of Zhang et al, 2023 Nature Comms. "Host heparan sulfate promotes ACE2 super-cluster assembly and enhances SARS-CoV-2-associated syncytium formation". These authors also use PIXN and MTN compounds and define different mechanisms based on ACE2 clustering for virus entry. The authors should mention this work in the Discussion and try to reconcile the different findings.

As suggested, we include the following discussion in the revised manuscript.

“Consistent with this possibility, HS may promote spike-dependent ACE2 super-cluster assembly at the cell surface and enhance SARS-CoV-2–associated syncytium formation, suggesting that HS may organize ACE2 nanoscale architecture in a cell–cell fusion context [43].”

(2) The authors should strengthen their case for the validity of HS-virion interactions as a therapeutic target by mentioning studies showing effectiveness of interference with HS-Covid interactions by heparin and other investigational drugs eg. first study to demonstrate heparin inhibition of SARS CoV2 attachment, Mycroft-West et al, Thromb Haemostatis, 2020; recent report of successful clinical trials of nebulized heparin, The Lancet, Sept 2025; and the superior efficacy of HS mimetic Pixatimod/PG545 compared to heparin (Guimond et al 2022 ACS Chemical Sciences).

We thank the reviewer for this suggestion and add the following paragraph with citations the reviewer mentioned in the Discussion section.

“Interfering with HS binding has been suggested as a therapeutic strategy to prevent and treat many viral infections that depend on HS for entry, including COVID-19 [1, 2, 9, 12]. Supporting this strategy, disrupting Spike–HS interactions, including inhibition by heparin and related glycans, reduces SARS-CoV-2 attachment/entry [51]. Clinical evaluation of inhaled/nebulized unfractionated heparin has reported improved clinical outcomes without major bleeding signals, supporting the feasibility of targeting airway-surface HS interactions [52]. HS mimetics, such as pixatimod (PG545), inhibit SARS-CoV-2 infection and exhibit greater potency than heparin in assays measuring inhibition of Spike/ACE2 engagement and viral infectivity [53]. These reports support the translational potential of therapeutically interfering with virion–HS binding.”

(3) Figure 1a: incorrect label for antibody.

Corrected, thank you.

(4) Some misspellings noted in the manuscript, e.g., MINFLLUX, so please recheck the manuscript for typos.

We have rechecked the manuscript and corrected the typos.
